# Warming-induced changes of broccoli head to cauliflower-like curd in *Brassica oleracea* are regulated by DNA methylation as revealed by methylome and transcriptome co-profiling

**DOI:** 10.1186/s43897-022-00047-8

**Published:** 2022-12-22

**Authors:** Zilei Yao, Lu Yuan, Ke Liu, Tingjin Wang, Bin Liu, Yan Zhao, Susheng Gan, Liping Chen

**Affiliations:** 1 Department of Horticulture, College of Agriculture and Biotechnology, Zhejiang University, Hangzhou, 310058 China; 2 Plant Biology Section, School of Integrative Plant Science, Cornell University, Ithaca, NY 14853 USA; 3 Present address: College of Agronomy and Biotechnology, Yunnan Agricultural University, Kunming, 650201 China

**Keywords:** *Brassica oleracea*, DNA methylation, Floral development cessation, Global warming, Heat stress

## Abstract

Increasingly warming temperature impacts on all aspects of growth and development in plants. Flower development is a complex process that is very sensitive to ambient temperature, and warming temperatures often lead to abnormal flower development and remarkably reduce the quality and yield of inflorescent vegetables and many other crops, which can be exemplified by *Brassica oleracea* cv. Green Harmony F1, a broccoli cultivar, whose floral development is ceased at inflorescence meristem (at 28 °C) or floral primordium stage (at 22 °C), forming a cauliflower-like curd (28 °C) or intermediate curd (22 °C) instead of normal broccoli head at 16 °C. However, the underlying molecular regulatory mechanisms are not well understood. Here we report that warming temperature (28 °C or 22 °C) induced hypermethylation of the genome, especially the promoter regions of such sets of genes as ribosome biogenesis-related and others, leading to the suppression of the apex-highly-expressed distinctive genes, subsequently resulting in the abnormal floral development, as revealed by methylome and transcriptome co-profiling. The regulation of warming-induced abnormal floral development in broccoli was further verified by the fact that the DNA methylation inhibitor 5-azacytidine (5-azaC) released the expression of genes from the warming temperature-induced suppression, and restored the broccoli development to normalcy at warming temperature. The research provided new approaches to breeding broccoli and other crops for growing in wider or warmer temperature zones.

**Supplementary Information:**

The online version contains supplementary material available at 10.1186/s43897-022-00047-8.

**Figure:**
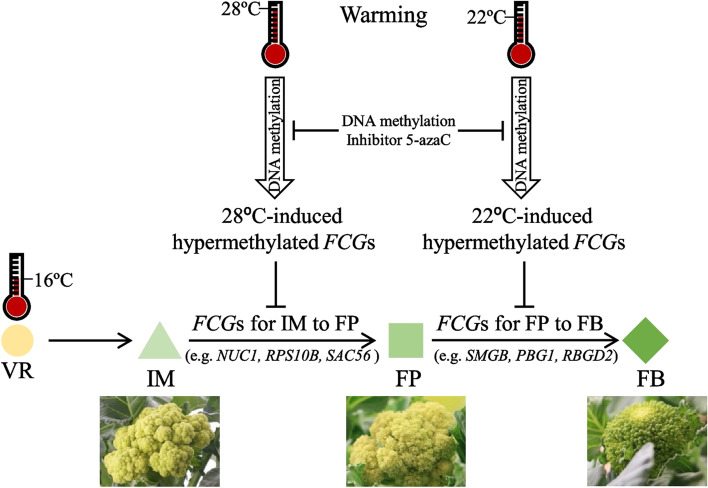
Graphical Abstract

## Core

Methylome and transcriptome co-profiling and treatment with DNA methylation inhibitor 5-azaC revealed that warming-induced abnormal floral development in broccoli was regulated by sets of apex-highly-expressed floral development cessation-associated genes (*FCG*s); warming caused hypermethylation of *FCG* promoters that suppressed *FCG*s’ expression. It provided new approaches to making crops adapt to increasingly warming environment.

## Gene and accession numbers

The WGBS and RNA sequencing data that support the findings of this study are available in Sequence Read Archive with accession number PRJNA897025 (for WGBS) and PRJNA897022 (for RNA-seq). All the other data generated in this study are included in the article and the additional files.

## Introduction

The elevating temperature on earth over decades of time due largely to the increased consumption of fossil energy causes climate change and threatens various forms of life on earth. Warming temperature impacts on all aspects of plant growth and development through their life cycle, starting from seed development and germination, slow vegetative growth, low photosynthesis and precocious senescence, and altered or abnormal flowering and reproductive development (Lippmann et al. 2019), which often results in significant reduction in crop yield and/or quality (Hedhly et al. 2009). Generally, flower development is more sensitive to high temperature than vegetative growth. For instance, heat stress applied at the vegetative stage had little effect on the biomass accumulation of wheat, but dramatically reduced the yield when applied at anthesis (Wollenweber et al. 2003). Similarly, the fecundity of *Arabidopsis* reduced remarkably when exposing to high temperature at intermediate and late flowering stage, compared to that when exposing at the vegetative stage (Scheepens et al. 2018). Therefore, it is essential to elucidate the regulation of warming temperature on floral development for improvement of crop yield and/or quality.

In crucifers including *Arabidopsis* flower development encompasses transition of vegetative shoot apical meristem to inflorescence meristem, the production of floral meristems, and the formation of an individual flower (Kwiatkowska 2006). *Brassica oleracea* is a species containing many varieties with different curd phenotypes, including cauliflower and broccoli head that result from cessations at different stages of floral development. The curd of cauliflower is composed of inflorescence meristems, and the curd of broccoli is consisting of floral buds. High temperature delayed the meristem transition and significantly increased the expression of *BoFUL-c* in the shoot apical meristem (SAM) of cauliflower plants (Sun et al. 2018). Heat stress also caused the cessation of broccoli floral development at an earlier stage, leading to uneven broccoli head (Bjorkman et al. 1998). Under heat stress, 24 miRNAs were differentially expressed during head formation in broccoli (Chen et al. 2015), and the expression of various meristem identity genes, including MADS-box genes (*BoAP1-a*, *BoAP1-c*, *BoCA*L, *BoFUL-a*, *BoFUL-b*, *BoFUL-c*, and *BoFUL-d*) and non-MADS-box genes (*BoLFY*, *AP2*, *UFO*, and *BoTFL1*) (Duclos et al. 2008), appeared to be irrelevant to the warming temperature-induced cessations of floral development in broccoli. The molecular regulatory mechanism underlying warming temperature-induced floral development cessations remains elusive.

We have been interested in understanding the molecular mechanism of warming temperature-induced abnormal floral development in plants. In this research, we chose Green Harmony F1 to address this question. Green Harmony F1 is a broccoli cultivar that is highly sensitive to ambient temperature. Warmer temperature causes the cultivar to develop into cauliflower-like curd (at 28 °C) or intermediate curd (22 °C) comparing the normal broccoli head at 16 °C (Duclos et al. 2008). Application of the DNA methylation inhibitor 5-azacytidine (5-azaC) eliminated the 28 °C-induced floral cessation at the inflorescence meristem stage (forming cauliflower-like curd), and the broccoli flower developed normally as if it were grown under 16 °C, suggesting that DNA methylation is involved in the warming temperature-induced floral cessations. Methylome and transcriptome co-profiling was thus utilized to unravel the underlying regulatory mechanism of warming on floral cessations. We found that warming induced global DNA hypermethylation, especially in the promoter regions of sets of genes (including apex-highly-expressed ribosome biogenesis-related genes), which suppressed the expression of these genes, and subsequently caused the floral development cessations.

## Results

### Warming temperature leads to cessation at different floral developmental stages in broccoli

**Fig. 1:**
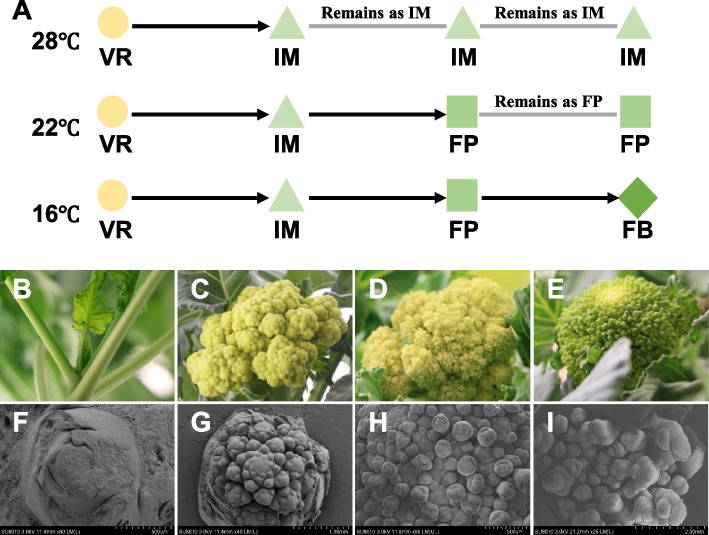
Effect of warming temperature on the floral development in broccoli (cv. Green Harmony F1). **A** Schematic diagram of floral development cessation caused by warming temperature in the broccoli cultivar. At 28 °C the floral development is ceased at the IM stage (forming cauliflower-like curd), at 22 °C the floral development is ceased at the FP stage (forming intermediate curd), while at 16 °C the flower develops into FB, forming normal broccoli head. Appearance (**B-E**) and scanning electron micrographs (**F-I**) of the apical meristems are shown. **B**,** F** shoot apical meristem at the transition from vegetative growth to reproductive growth (VR); (**C**, **G**) floral development ceased at the inflorescence meristem (IM) stage (cauliflower-like curd) at 28 °C; (**D**, **H**) floral development ceased at floral primordium (FP) stage (intermediate curd) at 22 °C; (**E**, **I**) floral development at floral bud (FB) stage (normal broccoli head) at 16 °C

Green Harmony F1, a broccoli cultivar, is known to be highly sensitive to elevated temperature, especially in terms of broccoli head development (Duclos et al. 2008). We are interested in understanding the molecular mechanisms underlying the warming-induced floral development cessation in this broccoli cultivar. Plants were grown under three temperature regimes: 28 °C, 22 °C, and 16 °C, and the floral development of the plants were ceased at inflorescence meristem stage (IM; producing cauliflower-like curd at 28 °C), floral primordium stage (FP; intermediate curd at 22 °C) or developed into floral bud (FB; normal broccoli head that is harvested and consumed) and beyond at 16 °C (Fig. 1A). At the transition from vegetative growth to reproductive growth (VR) stage, the apical meristem became flat and wide, the leaf primordium ceased to divide, and the bract leaf began to differentiate (Fig. 1B, F). Subsequently the apical meristem will give rise to the secondary, tertiary and higher order of inflorescence meristems, resulting in a thick curd. The development of the apical meristem at 28 °C was ceased at the IM stage (cauliflower-like curd) (Fig. 1C, G). At 22 °C, there were inflorescences branching out of the periphery of inflorescence meristem, that soon differentiated into floral primordia, forming intermediate curd (Fig. 1D, H). In contrast, at 16 °C the floral primordia rapidly developed into floral buds and formed broccoli head after brief curd-thickening (Fig. 1E, I).

### DNA methylation inhibitor eliminates warming temperature-induced floral development cessation

**Fig. 2:**
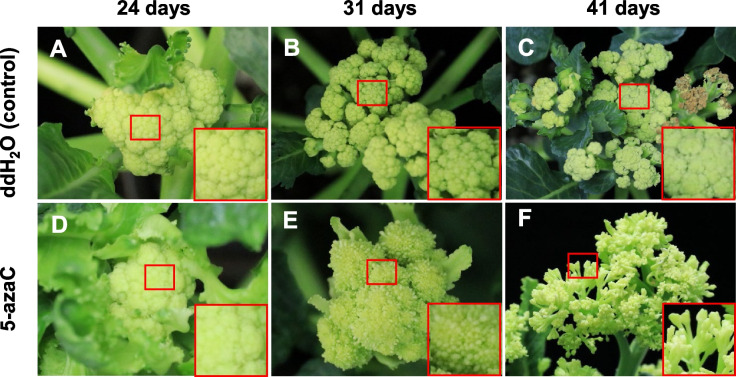
Elimination of 28 °C-induced floral development cessation at IM stage (cauliflower-like curd) by DNA methylation inhibitor 5-azaC in broccoli. **A-C** Floral development ceased at the inflorescence meristem stage (IM, cauliflower-like curd) in plants treated with ddH_2_O (control). **D-F** The inflorescence meristem (IM, panel **D**) developed into the floral primordia (FP; panel **E**) and floral buds (FB, panel **F**) in plants treated with the DNA methylation inhibitor 5-azaC. The red squares indicate zoomed view of the apex

We hypothesized that DNA methylation might be involved in the cessation of the floral development described above. We thus treated the plants at the VR stage grown at 28 °C with 5-azaC-treated and ddH_2_O (control), respectively. Twenty-four days after the treatment, the apical meristems of both the 5-azaC plants and the control developed cauliflower-like curd (IM stage) (Fig. 2A, D). Thirty-one days after the treatment, the floral development of the control plants remained the cauliflower-like curd, that is, the development was ceased at the IM stage (Fig. 2B). In contrast, the IM of the 5-azaC-treated plants differentiated into the intermediate curd or FP (Fig. 2E). Fourty-one days after the treatment, the intermediate curd continued developing and formed the broccoli head (Fig. 2F) while the control remained ceased at the IM stage (Fig. 2C). The results strongly suggested that the floral development cessation caused by warming temperature is regulated by DNA methylation.

### Global dynamics in DNA methylation under warming temperature regimes

The above results prompted us to investigate the global changes in DNA methylation during floral development at different temperature (28 °C, 22 °C, and 16 °C). Apexes at four development stages, VR, IM, FP, and FB, at different temperature as described in Fig. 1 were sampled and subjected to whole genome bisulfite sequencing, with two biological replicates. There were approximately 64.9 million clean reads for each sample, and more than 53% of the reads could be mapped to the *Brassica oleracea* reference genome (Supplementary Table S1). We identified 17,769,861 (28 °C), 16,564,204 (22 °C) and 16,384,000 (16 °C) methylated cytosines (mC) on average, the numbers of mC sites at 28 °C was significantly larger than those at 22 °C and 16 °C (Supplementary Table S2).

**Fig. 3:**
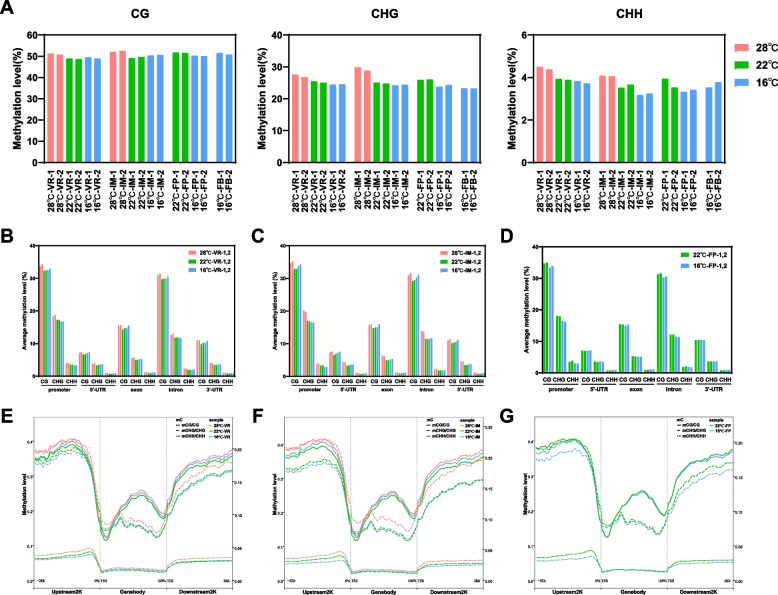
Global changes of genomic methylation levels during broccoli floral development under different temperature regimes. **A** Global genomic DNA methylation levels at different floral development stages (VR, IM, FP, FB as described in Fig. 1) under three temperature regimes in the CG, CHG and CHH contexts, respectively. Data from two biological replicates of whole-genome bisulfite-sequencing are presented. The different temperature regimes (28 °C, 22 °C and 16 °C) are shown in pink, green, and blue, respectively. **B-D** The average methylation levels of different genic regions in three sequence contexts (CG, CHG, and CHH) at different development stages (VR, IM, FP) under three temperature regimes, respectively. **E-G** Methylation profiles in protein-coding genes in the gene body and 2-kb flanking regions at the VR, IM, FP stage, respectively. The mean methylation levels of each 50-bp interval are plotted. TSS stands for transcriptional start site, and TES for transcriptional end site

Analyses of the global DNA methylation levels at different stages under the three temperature regimes (Supplementary Table S3) revealed that the methylation levels were higher at 28 °C than at 22 °C and 16 °C in all contexts (CG, CHG, and CHH; H represents A, T or C). For examples, the average methylation levels in the CG context at the VR stage increased from 49.2% at 16 °C to 51.0% at 28 °C; at the IM stage it increased from 50.5% at 16 °C to 52.3% at 28 °C; and at the FP stage from 50.1% at 16 °C to 51.7% at 22 °C (Fig. 3A). Similarly, there was an increase in the global methylation levels in the CHG and CHH contexts at the high temperature regimes (Fig. 3A).

The DNA methylation profiles of coding genes (including gene body and their 2-kb flanking regions) at different temperature (Fig. 3E through G) also showed that higher temperature led to higher methylation levels in the upstream and downstream regions of genes in all contexts (CG, CHG and CHH, especially CHG). The DNA methylation levels in different genic regions were further analyzed. The average methylation levels in the promoter regions increased with the increasing temperature (from 16 °C, 22 °C to 28 °C) in all contexts (CG, CHG, and CHH) (Fig. 3B through D). For instances, in the CG context, the average methylation levels of promoter regions increased from 32.8% at 16 °C to 34% at 28 °C at the VR stage, from 34% at 16 °C to 35% at 28 °C at the IM stage, and from 33.6% at 16 °C to 34.9% at 22 °C at the FP stage. Similarly, the average methylation levels of the promoter regions in the CHG context at the VR stage increased from 16.7% at 16 °C to 18.5% at 28 °C, at the IM stage from 16.5% at 16 °C to 19.9% at 28 °C, and at the FP stage from 16.3% at 16 °C to 18% at 22 °C. In the CHH context, the average methylation levels of the promoter regions at the VR stage increased ~ 0.8% from 16 °C to 28 °C, at the IM stage it increased about 1% from 16 °C to 28 °C, and at the FP stage, it increased approximately 0.7% from 16 °C to 22 °C. These data indicated that warming temperatures led to global increases in DNA methylation levels, especially in the promoter regions.

**Fig. 4:**
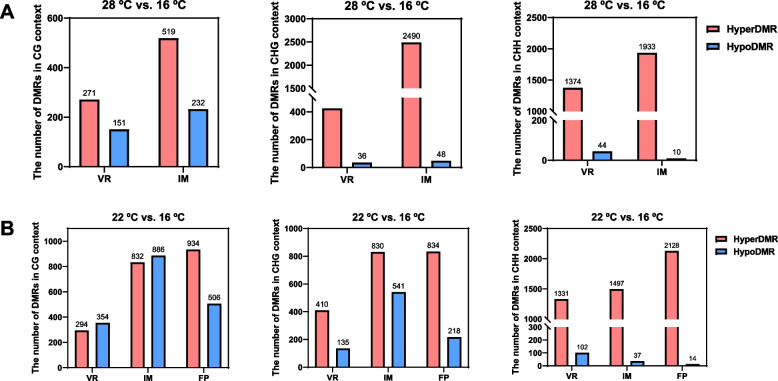
Genome-wide changes in warming temperature-induced differentially methylated regions (DMRs). **A** The numbers of hypermethylated and hypomethylated DMRs in the CG, CHG or CHH context at the same floral developmental stage (VR or IM) in the 28 °C vs. 16 °C comparison group. Hyper-DMR, hypermethylated DMR; Hypo-DMR, hypomethylated DMR. **B** The numbers of hypermethylated and hypomethylated DMRs in the CG, CHG or CHH context at the same floral developmental stage (VR, IM or FP) in the 22 °C vs. 16 °C comparison group

To investigate the role of DNA methylation in regulating warming temperature-induced floral development cessation, we further analyzed the DNA methylomes in two pairwise comparison groups: 28 °C vs. 16 °C and 22 °C vs. 16 °C. Seven thousand, five hundred thirty-two differentially methylated regions (DMRs) in group of 28 °C vs. 16 °C were identified with 7011 hypermethylated (hyper-DMRs) and 521 hypomethylated (hypo-DMRs) (Fig. 4A). The numbers of hyper-DMRs in CHG and CHH contexts were also larger than those of hypo-DMRs in the group of 22 °C vs. 16 °C (Fig. 4B). These results collectively indicated that warming temperature caused the increases in genome-wide DNA methylation levels in general, and the numbers of hyper-DMRs in particular (although there were also hypo-DMRs).

### Warming temperature alters gene expression patterns during floral development

**Fig. 5:**
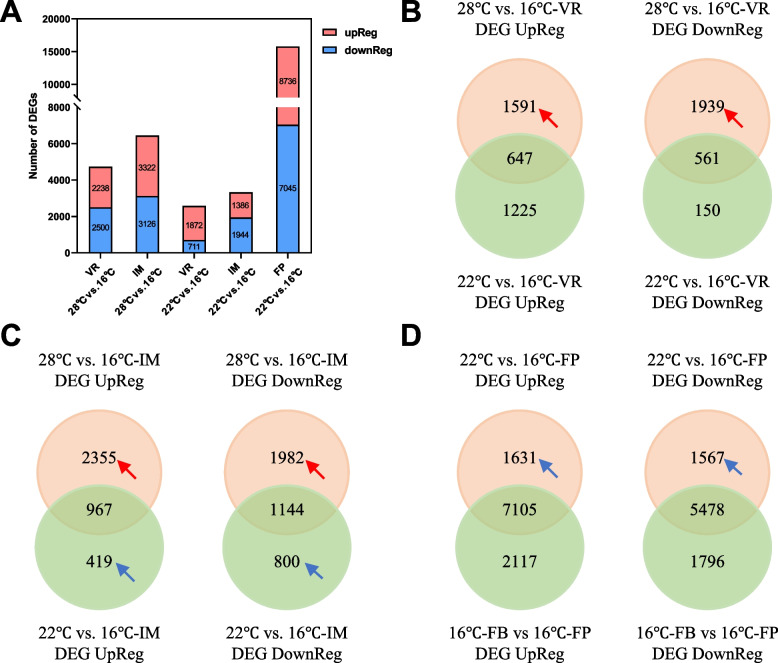
Numbers of up- and down-regulated differentially expressed genes (DEGs) induced by warming temperature. **A** Numbers of up- and down-regulated DEGs in two comparison groups (28 °C vs. 16 °C and 22 °C vs. 16 °C). **B-D** Venn diagrams showing overlaps among up- and down-regulated DEGs at the VR, IM, FP stage, respectively. For the FP stage, the DEGs between 22 °C -FP vs. 16 °C -FP and 16 °C -FB vs. 16 °C -FP were compared; by doing so is to exclude those common genes that function in floral development rather than cessation. The DEGs were determined using DESeq2 with FDRs < 0.05, estimated by the adjusted *p*-value. The red arrows indicate DEGs that are potentially involved in floral cessation at the IM stage. The blue arrows indicate DEGs that are potentially involved in floral cessation at the FP stage

To determine whether the DNA methylation contribute to changes in gene expression during floral development, transcriptomes at different stages of floral development under various temperature regimes were generated, with three biological replications. After data filtering, approximately 87% of reads were uniquely mapped to the *B. oleracea* reference genome (Supplementary Table S4). We identified differentially expressed genes (DEGs) by pairwise analyses among three temperature regimes (28 °C, 22 °C and 16 °C) with emphases on two groups of them (28 °C vs. 16 °C; 22 °C vs. 16 °C). Comparing the transcriptomes at 28 °C vs. 16 °C led to the identification of 2238 up- and 2500 down-regulated DEGs at the VR stage, and 3322 up- and 3126 down-regulated DEGs at the IM stage (Fig. 5A). Similarly, comparison of the transcriptomes at 22 °C vs. 16 °C resulted in the identification of 1386 up- and 1944 down-regulated DEGs at the IM stage, and 8736 up- and 7045 down-regulated DEGs at the FP stage. These results clearly showed that the warmer temperatures remarkably altered the gene expression dynamics during floral development.

There were overlapping DEGs between “28°C vs. 16°C” and “22°C vs. 16°C” at all floral development stages as revealed by Venn analysis (Fig. 5B through D). It is reasonable to hypothesize that the non-overlapping DEGs found at and/or before a specific floral cessation stage may most likely be involved in the warming temperature-induced floral cessations, we thus focused our further analyses on the non-overlapping DEGs. For instance, the floral development was ceased at the IM stage at 28 °C, but not at 22 °C (Fig. 1), and the non-overlapping 2355 up- and 1982 down-regulated DEGs identified at the IM stage from 28 °C vs. 16 °C were most likely responsible for the floral development cessation at the IM stage (Fig. 5C). The non-overlapping 1591 up- and 1939 down-regulated DEGs identified at the VR stage from 28 °C vs. 16 °C might also be responsible for the cessation at the IM stage (Fig. 5B). Similarly, the non-overlapping 1631 up- and 1567 down-regulated DEGs identified at the FP stage from 22 °C vs. 16 °C were most likely responsible for the floral development cessation at the FP stage (Fig. 5D), and the non-overlapping 419 up- and 800 down-regulated DEGs identified at the IM stage from 22 °C vs. 16 °C might be responsible for the cessation at the FP stage as well (Fig. 5C).

### Warming temperature-induced hypermethylation regulates apex-highly-expressed genes such as those ribosome biogenesis-related genes

**Fig. 6:**
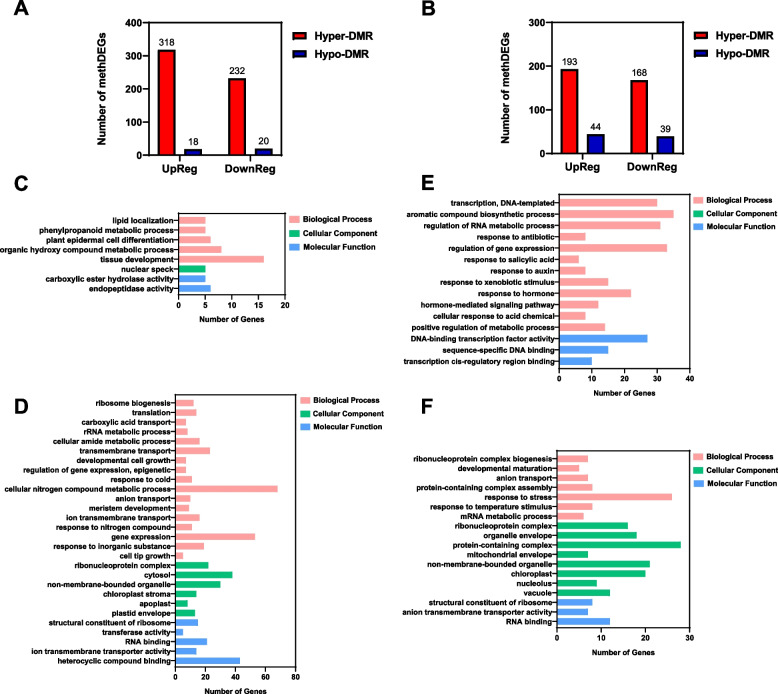
Gene Ontology (GO) term analysis of differentially-methylated DEGs (methDEGs) associated with floral development cessations. **A-B** Numbers of methDEGs associated with 28 °C-induced floral cessation at the IM stage (**A**) or 22 °C-induced floral cessation at the FP stage (**B**). **C-D** GO enrichment of up- (**C**) and down-regulated (**D**) methDEGs at the 28 °C-induced floral cessation at the IM stage. **E-F** GO term analysis of up- (**E**) and down-regulated (**F**) methDEGs at the 22 °C-induced floral cessation at the FP stage

We hypothesized that a subset of the above identified DEGs potentially responsible for the floral development cessation at the IM stage or FP stage might be regulated by DNA methylation. We thus performed association analyses between DMRs and DEGs, and identified a number of differentially-methylated DEGs (methDEGs) that may be involved in the floral development cessations. For the floral cessation at the IM stage at 28 °C, 318 up- and 232 down-regulated genes were hypermethylated, and 18 up- and 20 down-regulated genes hypomethylated (Fig. 6A). For the floral cessation at the FP stage (22 °C), 193 up- and 168 down-regulated genes were hypermethylated, and 44 up- and 39 down-regulated genes hypomethylated (Fig. 6B). For both cessations (IM and FP) hypermethylated genes, up- or down-regulated, were predominant.

Gene Ontology (GO) analyses of these methDEGs revealed that for the 28 °C-induced floral cessation at the IM stage, genes involved in expression, ribosome biogenesis and transmembrane transport were abundant among the down-regulated methDEGs, while 16 of the up-regulated methDEGs were responsible for tissue development (Fig. 6C, D). Further analyses of individual genes with their counterpart genes in Arabidopsis revealed that some methDEGs are highly expressed in inflorescence meristem; examples of these methDEGs are *LOC106315696* (counterpart in Arabidopsis *AT1G60080*, a 3–5-exoribonuclease family protein), *LOC106294921* (*AT2G34480*, *RPL18AB*, a nuclear localized member of the ribosomal L18ae/LX protein family), *LOC106300910* (*AT1G48920*, *NUC1*, a nucleolin protein involved in rRNA processing, ribosome biosynthesis, and vascular pattern formation), *LOC106331732* (*AT4G33250*, *EIF3K*, the initiation factor 3 k), *LOC106298457* (*AT5G41520*, *RPS10B*, the eukaryote-specific protein S10e of the small cytoplasmic ribosomal subunit), and *LOC106336129* (*AT3G09630*, *SAC56*, a ribosomal protein L4/L1 family) (Supplementary Fig. S1, Supplementary Table S5). The subset of the methDEGs identified here are highly expressed in the shoot apex and most likely responsible for the floral development cessation at the IM stage under 28 °C, which are designated as floral development cessation-associated genes (*FCG*s). I.e., these *FCG*s are required for the floral development from IM to FP (*FCG*s for IM to FP).

For the 22 °C-induced floral cessation at the FP stage, the up-regulated methDEGs were rich in genes involved in RNA metabolic process, transcription, and response to hormones while the down-regulated methDEGs were well represented by genes involved in response to stress, ribonucleoprotein complex, and RNA binding (Fig. 6E, F). In inferring from the counterpart genes in Arabidopsis, a number of methDEGs were found to be highly expressed in the shoot apex inflorescence: *LOC106336011* (*AT5G02960*, a ribosomal protein S12/S23 family protein), *LOC106316876* (*AT5G48870*, *SAD1*, a polypeptide similar to multifunctional Sm-like snRNP proteins that are required for mRNA splicing, export, and degradation), *LOC106315720* (*AT5G20920*, the protein synthesis initiation factor eIF2 beta), *LOC106313474* (*AT5G19510*, the translation elongation factor EF1B), *LOC106337101* (*AT3G53870*, a ribosomal protein S3 family protein), and *LOC106342054* (*AT1G56450*, *PBG1*, 20S proteasome β subunit) (Supplementary Fig. S1, Supplementary Table S5). These methDEGs or *FCG*s are required for the floral development from FP to FB (*FCG*s for FP to FB).

### The tripartite relation among temperature, methylation levels and transcript abundance of individual *FCG*s

**Fig. 7:**
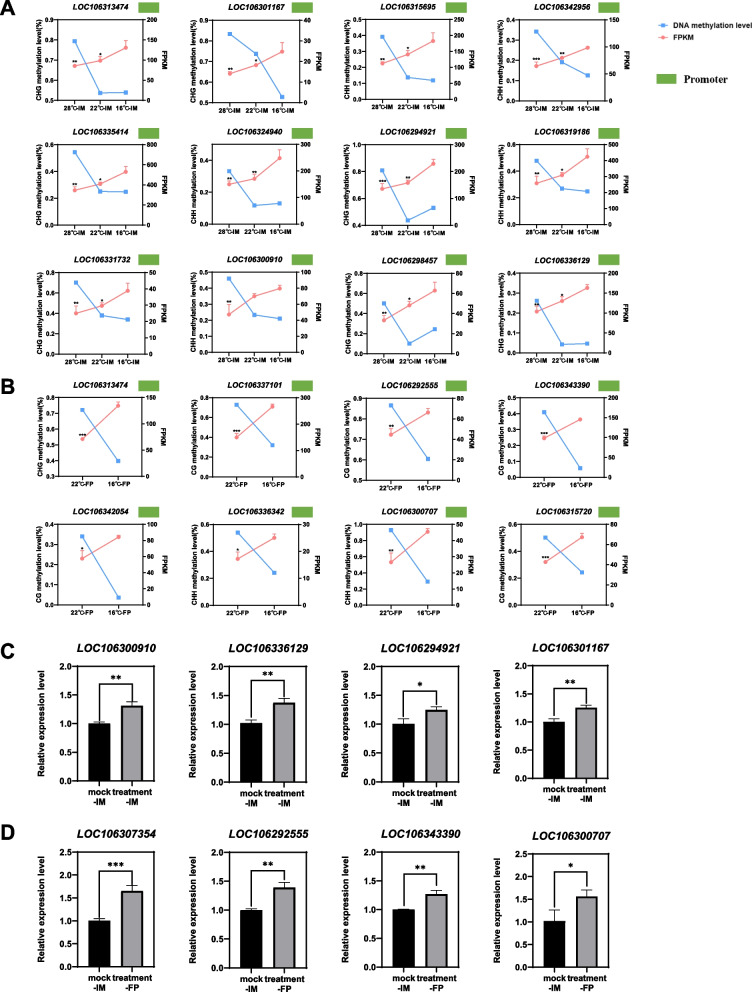
Effects of temperature and DNA methylation inhibitor on DNA methylation levels and transcript levels of *FCG*s. **A-B** Tripartite relation among temperature, DNA methylation levels and transcript levels of some *FCG*s likely responsible for the 28 °C-induced floral cessation at the IM stage (**A**), and the 22 °C-induced floral cessation at the FP stage (**B**). A methylation level is the average of two biological replicates; and a transcript level represents the mean of a gene’s transcript levels present in three related transcriptomes. The green blocks indicate promoter region. Error bars indicate means ± SD. **C-D** qPCR analyses of randomly selected *FCG*s in mock and methylation inhibitor 5-azaC treated plants at the IM stage (**C**) and FP stage (**D**). Asterisks indicate significant differences relative to 16 °C (**A**, **B**), ANOVA analysis with Dunnett’s test (**A**) and T-test (**B**, **C**) were used. *p < 0.05, **p < 0.01, ***p < 0.001

The above co-profiling of methylomes and transcriptomes at warming temperature enabled the identification of *FCG*s that might be responsible for the floral cessations. We further examined the methylation levels and expression levels of individual *FCG*s at different temperatures, which revealed a positive relation between methylation levels and temperature, and a negative relation between the methylation levels and transcript abundances of *FCG*s (Fig. 7A, B). That is, the increasing temperature increased the methylation levels of *FCG*’s promoters, and reduced the *FCG*’s transcript levels, supporting the hypothesis that warming temperature induced hypermethylation, which in turn suppressed *FCG*s’ expression, resulting in the floral cessation at IM or FP.

### The warming temperature-induced hypermethylation is the cause of the suppression of *FCG*s’ expression

To investigate that the warming temperature-induced hypermethylation was responsible for the suppression of *FCG*s’ expression revealed above, we performed real-time quantitative PCR (qRT-PCR) analysis of randomly selected *FCG*s from samples treated with or without the DNA methylation inhibitor 5-azaC at warming temperature. The expression levels of these *FCG*s in samples treated with the methylation inhibitor were significantly higher than those in controls at 28 °C or 22 °C (Fig. 7C, D), confirming that the warming temperature-induced DNA hypermethylation is the cause of the suppression of *FCG*s.

### The DMRs on the *FCG* promoters are transcription factor (TF) binding sites

**Fig. 8:**
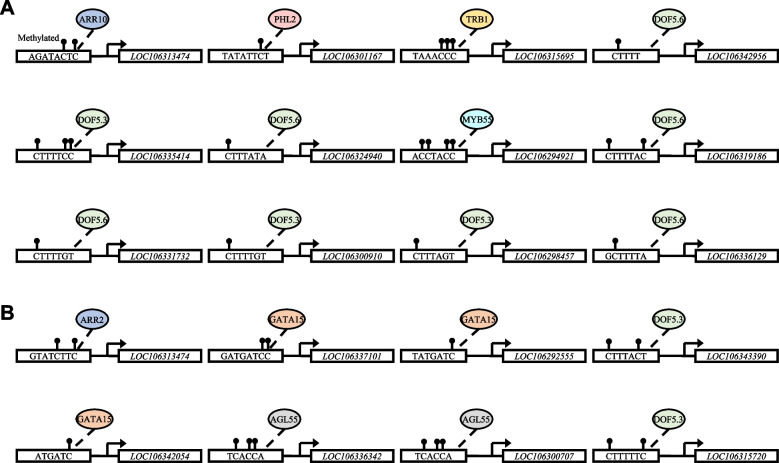
Predicted transcription factors that bind to the DMRs of individual *FCG* promoters. Warming temperature (28 °C or 22 °C) causes DNA hypermethylation in the promoter regions of some *FCG*s shown in Fig. 7. Predicted transcription factors (represented by ellipses) using JASPAR^2022^ (https://jaspar.genereg.net) are potentially binding to the DMRs on the promoters of *FCG*s responsible for 28 °C-induced floral cessation at the IM stage (**a**) or 22 °C-induced floral cessation at the FP stage (**b**). The ellipses with the same color represent identical or very similar transcription factors

The findings that DNA hypermethylation suppressed gene expression as described above prompted us to investigate whether the DMRs on the *FCG* promoters are TF binding sites; the hypermethylation may prevent TF bindings. We used DMR sequences of the promoters of the *FCG*s in Fig. 7 to search against JASPAR^2022^ database; the JASPAR CORE database contains a curated, non-redundant set of profiles, derived from published collections of experimentally defined transcription factor binding sites for eukaryotes (https://jaspar.genereg.net). All the DMRs were predicted to be *cis* elements to which various TF binds (Fig. 8A, B). Among the TFs binding to the promoters of the *FCG*s responsible for 28 °C-induced floral cessation are ARR10 (a type-B ARR TF involved in the cytokinin signaling), PHL2 (a MYB-CC protein involved in regulation of response to phosphate starvation), TRB1 (telomere repeat binding factor 1, functioning together with MYB domain), MYB55 and DOFs (DNA-binding one finger), and the DOF family TFs are predominant (Fig. 8A). In contrast, ARR2 (also a TF involved in the cytokinin signaling), GATA15 (a Zinc finger TF involved in cytokinin response), AGL55 (AGAMOUS-like 55 TF highly expressed in shoot apical meristem) and DOFs are among the TFs binding to the promoters of the *FCG*s responsible for 22 °C-induced floral cessation (Fig. 8B).

## Discussion

**Fig. 9.**
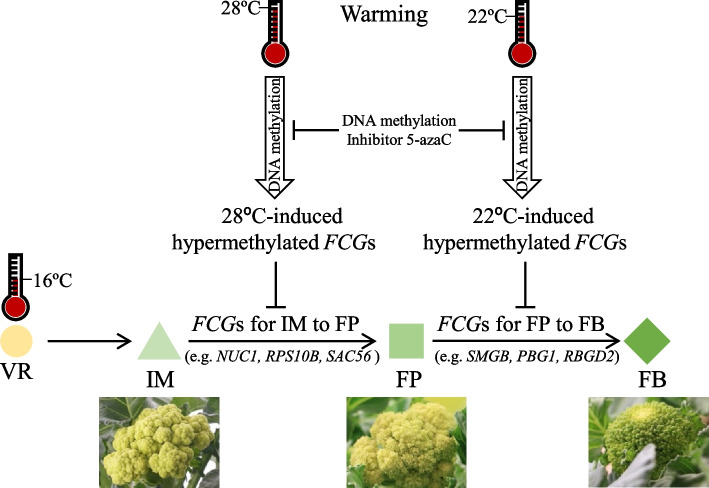
A proposed model illustrating molecular and epigenetic mechanism underlying warming-induced floral cessations in broccoli (cv. Green Harmony F1). At 16 °C the broccoli flower will develop through VR, IM, FP to FB to produce normal broccoli head. Warming (28 °C or 22 °C) causes genome DNA hypermethylation, especially in the promoter regions of floral development cessation-associated genes (*FCG*s), which suppresses the expression of the apex-highly-expressed *FCG*s’ whose expression is required for the floral development from IM to FP (28 °C) or from FP to FB (22 °C), resulting in the floral cessations at the IM stage (28 °C; cauliflower-like curd) or at the FP stage (22 °C; intermediate curd). The DNA methylation inhibitor 5-azaC will block the DNA hypermethylation and eliminate the warming-induced floral cessations

It is well documented that increasingly warming temperature severely impacts on plant development, especially the reproductive growth stages (Li et al. 2013). The flower development of *Brassica* crops such as cauliflower and broccoli are very sensitive to elevated temperature and result in “bracting” in curds and other abnormal phenotypes (Bjorkman et al. 1998; Grevsen et al. 2015). Flower identity genes are unlikely involved in the abnormalcy (Duclos et al. 2008). The present research unraveled a novel regulatory mechanism underlying warming-induced floral cessations in broccoli. Briefly, warming causes global genomic hypermethylation, especially in the promoter regions of sets of genes called *FCG*s that are required for normal floral development, resulting in the suppression of the expression of the apex-highly-expressed *FCG*s and the cessations of floral development (Fig. 9).

There are two lines of evidence supporting that DNA hypermethylation is involved in the warming temperature-induced floral cessation. (i) The chemical 5-azaC eliminated the 28 °C-induced floral cessation at the IM stage (cauliflower-like curd) in the broccoli cv. Green Harmony F1 (Figs. 1 and 2). The chemical 5-azaC has been shown to effectively inhibit DNA methylation (Zhong et al. 2013). (ii) Whole genome bisulfite sequencing was performed to profile the dynamic changes in methylomes of the broccoli grown at various temperatures, which revealed that the DNA methylation levels in all contexts (CG, CHG, and CHH) were elevated with the increasing temperature (Fig. 3). DNA methylation occurs in three different cytosine sequence contexts: CG, CHG and CHH (Henderson et al. 2007) and plays a significant role in plant responses to temperature variation (Bossdorf et al. 2008). For example, the genome DNA methylation levels of Chinese cabbage (*Brassica rapa*) leaves increased under heat stress (Liu et al. 2018). It should be noted that the transcript levels of DNA methylase-related genes were not increased with the increasing temperature as revealed by analyzing the related transcriptomes (Supplementary Fig. S2), and the biochemical mechanism underlying the increased temperature-induced DNA hypermethylation is yet to be deciphered.

The warming temperature-induced genome hypermethylation in the promoter regions suppresses sets of *FCGs* (Fig. 9), which is supported by (i) association study between the methylation levels and transcript abundance of *FCGs* (Fig. 7A, B), (ii) qPCR analyses of *FCG*s’ expression with or without the DNA methylation inhibitor 5-azaC treatment (Fig. 7C, D), and (iii) DMRs-TF binding prediction (Fig. 8A, B). WGBS analysis led to the identification of DNA hypermethylation changes in single-base resolution. There are a wide range of hyper-DMRs induced by warming temperatures in two pairwise comparison groups, 28 °C vs. 16 °C and 22 °C vs. 16 °C (Fig. 4). Analyses of hyper-DMRs and related transcriptomes revealed that there was an inverse relation between the DNA methylation levels in the promoter regions and the gene expression of *FCG*s unique to 28 °C (Fig. 7A) or 22 °C (Fig. 7B). The inverse relation between methylation levels and gene transcript abundance is consistent with many other studies (Raza et al. 2017; Yu et al. 2018; Yuan et al. 2020) although DNA hypermethylation may activate gene expression (Huang et al. 2019; Lang et al. 2017). The DNA hypermethylation suppressed the related gene expression because the treatment with the DNA methylation inhibitor 5-azaC released the suppression of the randomly selected *FCG*s at 28 °C (Fig. 7C) or 22 °C (Fig. 7D). Furthermore, the DMRs on the promoters of the FCGs were all predicted to be the TF binding sites (Fig. 8A, B); hypermethylation of the sites prevented from TF binding, resulting in the reduction in gene expression.

*FCG*s are among methDEGs, and most likely responsible for normal broccoli floral development. When a set of *FCG*s unique to 28 °C is suppressed by the 28 °C-induced DNA hypermethylation, the broccoli floral development is ceased at the IM stage, forming cauliflower-like curd; similarly, when a set of *FCG*s unique to 22 °C is suppressed by the 22 °C-induced DNA hypermethylation, the broccoli floral development is ceased at the FP stage, forming intermediate curd (Fig. 9). GO enrichment analysis revealed that ribosome biogenesis- or RNA binding-related genes are rich among the methDEGs (Fig. 6C through F, and Supplementary Table S5). Ribosome biogenesis has been a hot research topic (Klinge et al. 2019). It plays a significant role in regulating cell growth and proliferation, and contributes to diseases such as cancer (Bohnsack et al. 2019). In plants, the heterogeneous yet specialized ribosomes regulate mRNA translation and control protein synthesis (Martinez-Seidel et al. 2020), and the ribosome biogenesis is correlated to the growth of meristematic cells (Baserga 2007). It is thus reasonable to hypothesize that the IM cessation- or FP cessation-specific *FCG*s are required in order to overcome the respective cessation during floral development in broccoli. More relevantly, the Arabidopsis counterparts of these *FCG*s are highly expressed in apex (Supplementary Fig. S1). For instance, *NUC1* (*LOC106300910*), encoding a nucleolin-like protein, plays a role in ribosome biogenesis, processing of pre-rRNA and cell growth (Manzano et al. 2021), and highly expressed in proliferating tissues (Pontvianne et al. 2007). *SAD1* (*LOC106316876*) encodes a polypeptide similar to multifunctional Sm-like snRNP proteins that control splice-site recognition and contributes to stress tolerance in Arabidopsis (Cui et al. 2014). *RPS10B* (*LOC106298457*), encoding the eukaryote-specific protein S10e of the small cytoplasmic ribosomal subunit, was found to promote shoot branching (Patzke et al. 2019; Stirnberg et al. 2012). The specific function of individual *FCG*s in warming-induced abnormal floral development requires further molecular genetic investigations. methDEGs other than ribosome biogenesis-related genes may also play a role in the warming-induced floral cessations, and deserve future study as well.

Lastly, this research provides new approaches to breeding of broccoli and other crops for resilience to elevating temperature or for cultivating in warmer temperature zones. Broccoli cultivars (and many other crops) are highly regional, and for example, the northeast region-well-grown cultivars (https://blogs.cornell.edu/easternbroccoliproject/main/production/varieties/) may hardly grow well in southern USA. Blocking DNA methylation chemically or genetically (via knocking out the related DNA methyltransferase gene(s) by genome editing) will allow these cultivars to be produced in the warmer regions. Enhancing the expression of *FCG*s under warmer temperature condition may also make these cultivars perform well in the southern or warmer regions.

## Materials and methods

### Plant material and growth conditions

Seeds of the *Brassica oleracea* (cv. Green Harmony F1), a hybrid of cauliflower and broccoli (Known-You Seed Company Ltd., Taiwan) were sown Petri dishes containing one-half strength Murashige and Skoog salts (Murashige et al. 1962). One-month-old seedlings were transplanted into soil mix (Lambert) and grown in growth chambers at day/night temperature of 23 °C /17 °C, with 14 h light/7 h dark photoperiod, 75% relative humidity, and light intensity of ~ 200 μmol·m^− 2^·s^− 1^. After 1 week of growth, the plants were moved to chambers with three different temperature settings: 28 °C, 22 °C, and 16 °C with photoperiod, relative humidity, and light intensity unchanged. Apex issues at VR, IM, FP, FB stages under three different temperature regimes were sampled for WGBS and RNA-seq analyses (Table 1).

**Table 1:** Materials harvested for methylomes and transcriptomes

Temperature	VR	IM	FP	FB
28 °C	√	√ (cessation)		
22 °C	√	√	√ (cessation)	
16 °C	√	√	√	√

For each sampling point (√), 2 biological replicates (thus 2 methylomes) and 3 biological replicates (3 transcriptomes) were used

### Scanning electron micrographs (SEM)

The apex tissues were first fixed with 2.5% glutaraldehyde in phosphate buffer (0.1 M, pH 7.0), washed three times with the phosphate buffer, then postfixed with 1% OsO_4_ in the buffer for 1–2 h and washed three times with the buffer; each wash last for 15 min. Next, the tissues were dehydrated by a series of ethanol and finally by using a Hitachi critical point dryer (Model HCP-2). The dehydrated samples were coated with gold-palladium using a Hitachi ion sputter (Model E-1010) for 4–5 min and checked using a Hitachi SEM (Model SU-8010).

### 5-azacytidine treatment

1.5 mL of 0.2 mM 5-azacytidine (Biorab, Beijing, China) were sprayed on apexes and leaves every day starting at the VR stage (3 weeks after transplanting), and ddH_2_O was sprayed as the mock control. The apexes were collected at the VR, IM, FP, and FB stages from the 5-azaC treatment group and at the VR and IM stages from the control group, respectively.

### Whole-genome bisulfite sequencing and data analysis

Total genomic DNA was separately extracted from individual samples (Table 1), followed by quality checks using a NanoPhotometer® spectrophotometer (IMPLEN, CA, USA). The DNA concentration was measured using Qubit® 2.0 Flurometer (Life Technologies, CA, USA) with a Qubit® DNA Assay Kit.

For library construction, DNA was fragmented into 200–300 bp with Covaris S220. EZ DNA Methylation-Gold™ Kit (Zymo Research) was subsequently used for bisulfite conversion. The quality of libraries was assessed using the Agilent Bioanalyzer 2100 system. Sequencing was performed by NovoGene Corporation (Beijing, China) using the Illumina platform (Illumina, CA, USA). FastQC (fastqc_v0.11.5) was used to assess the quality of the raw reads. After removing the adapters and low-quality reads with fastp (fastp 0.20.0), the remaining reads were counted as clean reads.

For data analysis, *Brassica oleracea* genome data (https://ftp.ncbi.nlm.nih.gov/genomes/all/GCF/000/695/525/GCF_000695525.1_BOL/) served as reference genome. Bismark software (version 0.16.3) (Krueger et al. 2011) was used to perform alignments of bisulfite-treated reads to a reference genome (−X 700 --dovetail). Both of the reference genome and the clean reads were transformed into bisulfite-converted version. Subsequently, alignment between converted versions of the reads and genome were performed in a directional manner. By comparing sequence reads with the normal genomic sequence, all cytosine methylation sites were mapped.

The binomial test was performed to identify each true methylated cytosine site using the methylated cytosine reads (mC), total cytosine reads (mC + umC) and the non-conversion rate (r), with a 0.05 FDR-corrected *p*-value. The sequence was divided into multiple bins of 10 kb, and the methylation level of the sequence was calculated using mC/(mC + umC), i.e., the number of methylated cytosine reads divided by the sum of methylated and unmethylated cytosine reads.

Differentially methylated regions (DMRs) were identified using the DSS software (Feng et al. 2014; Wu et al. 2015; Park et al. 2016). DSS is a new dispersion shrinkage method for estimating the dispersion parameter from Gamma-Poisson or Beta-Binomial distributions. According to the genomic distribution of DMRs, we defined a gene as DMR-associated genes (DMGs) if its gene body region (from transcription start site or TSS to transcription end site or TES) and/or its promoter region (2-kb upstream from TSS) overlap with a DMR or DMRs. DMGs were significantly enriched with GO seq R package (Park et al. 2016), with corrected *P*-value lower than 0.05. KOBAS software (Mao et al. 2005) was performed to test the statistical enrichment of DMG in KEGG pathways.

### RNA sequencing

Total RNA was extracted from the apexes with three biological replicates (Table 1). RNA degradation and contamination was validated by running 1% agarose gels. RNA purity was checked with the NanoPhotometer® spectrophotometer (IMPLEN, CA, USA). The RNA quality was confirmed using the RNA Nano 6000 Assay Kit of the Bioanalyzer 2100 system (Agilent Technologies, CA, USA).

Libraries were constructed using NEBNext® Ultra™ RNA Library Prep Kit for Illumina® (NEB, USA) according to manufacturer’s instruction. The library preparations were sequenced on an Illumina Novaseq platform and 150 bp paired-end reads were generated. Raw data (raw reads) of fastq format were firstly processed. Clean sequence data were obtained by removing adapter sequences, ploy-N and low-quality reads from raw data, and were used to calculate Q20, Q30 and GC content. Clean reads were mapped to the *Brassica oleracea* reference genome (https://ftp.ncbi.nlm.nih.gov/genomes/all/GCF/000/695/525/GCF_000695525.1_BOL/) using Hisat2 v2.0.5.

Gene expression was quantified by FPKM, which was defined as expected number of fragments per kilobase per million mapped reads. Subsequently, differential expression analysis was performed using the DESeq2 R package (1.16.1). To control the false discovery rate, Benjamini and Hochberg’s method was used to adjust the *P*-values. The threshold for differentially expressed genes (DEGs) were foldchange ≥1.5 and adjusted *P*-value ≤0.05. Gene Ontology (GO) terms and KEGG pathways enrichment analysis of DEGs were implemented using the cluster Profiler R package. GO terms and KEGG pathways with corrected *P*-value ≤0.05 were considered significantly enriched in DEGs.

### qRT-PCR

Total RNA was extracted from the apexes using an RNAprep pure Plant Kit (Tiangen Biotech, Beijing, China). cDNA was synthesized with HiScriptII QRT SuperMix Kit (Vazyme, China). qRT-PCR was performed using the Applied Biosystems StepOne™ Real-Time PCR System with the ChamQ SYBR qPCR Master Mix Kit (Vazyme, China). The primers are listed in Supplementary Table S6. For each sample, at least three replicates were performed. The relative transcript level of each gene was calculated using the 2^-ΔΔCT^ method normalized to the expression levels of *Actin* in the same samples.

### Supplementary Information

**Additional file 1: **The online version contains supplementary material available at (web address will be provided by the publisher). **Supplementary Fig. S1.** The snapshot of eFP browser results showing some of methDEGs that are highly expressed in shoot apex in *Arabidopsis*. **Supplementary Fig. S2.** Transcript levels of genes encoding DNA methyltransferases and demethylases in broccoli at three temperature regimes. **Supplementary Table S1.** Details of bisulfite sequencing libraries. **Supplementary Table S2.** The methylation density of whole genome. **Supplementary Table S3.** Methylation levels of whole-genome. **Supplementary Table S4.** Data from RNA-sequencing. **Supplementary Table S5.** List of floral development cessation-associated genes (*FCG*s). **Supplementary Table S6.** Primer sequences for qRT-PCR.

## Data Availability

All data are publicly accessible listed under “Gene and Accession Numbers”. Seeds of broccoli cultivar Green Harmony F1 is commercially available.

## Abbreviations

5-azaC: 5-azacytidine
AGL55: agamous-like 55
ARR: Arabidopsis response regulator
BSP: bisulfite sequencing PCR
DEG: differentially expressed gene
DMG: differentially methylated gene
DMR: differentially methylated region
DOF: DNA-binding one finger
*FCG*s: floral development cessation-associated genes
FP: floral primordium
FB: floral bud
GATA1: GATA-binding factor 1
GO: gene ontology
IM: inflorescence meristem
mC: methylated cytosine
MYB: myeloblastosis
PHL2: PHR1-LIKE 2
TES: transcription end site
TRB1: telomere repeat binding factor 1
TSS: transcription start site
VR: transition from vegetative growth to reproductive growth
umC: unmethylated cytosine
UTR: untranslated regions
WGBS: whole genome bisulfite sequencing
