# Supplementary material for: Warming-induced changes of broccoli head to cauliflower-like curd in Brassica oleracea are regulated by DNA methylation as revealed by methylome and transcriptome co-profiling
Source: Mol Hortic. 2022 Dec 22;2:26. doi: 10.1186/s43897-022-00047-8 (PMC10515005; doi:10.1186/s43897-022-00047-8)
Supplement: Supplementary file 1 — Additional file 1: The online version contains supplementary material available at (web address will be provided by the publisher). Supplementary Fig. S1. The snapshot of eFP browser results showing some of methDEGs that are highly expressed in shoot apex in Arabidopsis. Supplementary Fig. S2. Transcript levels of genes encoding DNA methyltransferases and demethylases in broccoli at three temperature regimes. Supplementary Table S1. Details of bisulfite sequencing libraries. Supplementary Table S2. The methylation density of whole genome. Supplementary Table S3. Methylation levels of whole-genome. Supplementary Table S4. Data from RNA-sequencing. Supplementary Table S5. List of floral development cessation-associated genes (FCGs). Supplementary Table S6. Primer sequences for qRT-PCR. [file 43897_2022_47_MOESM1_ESM.docx]

**Supplementary Information**

**
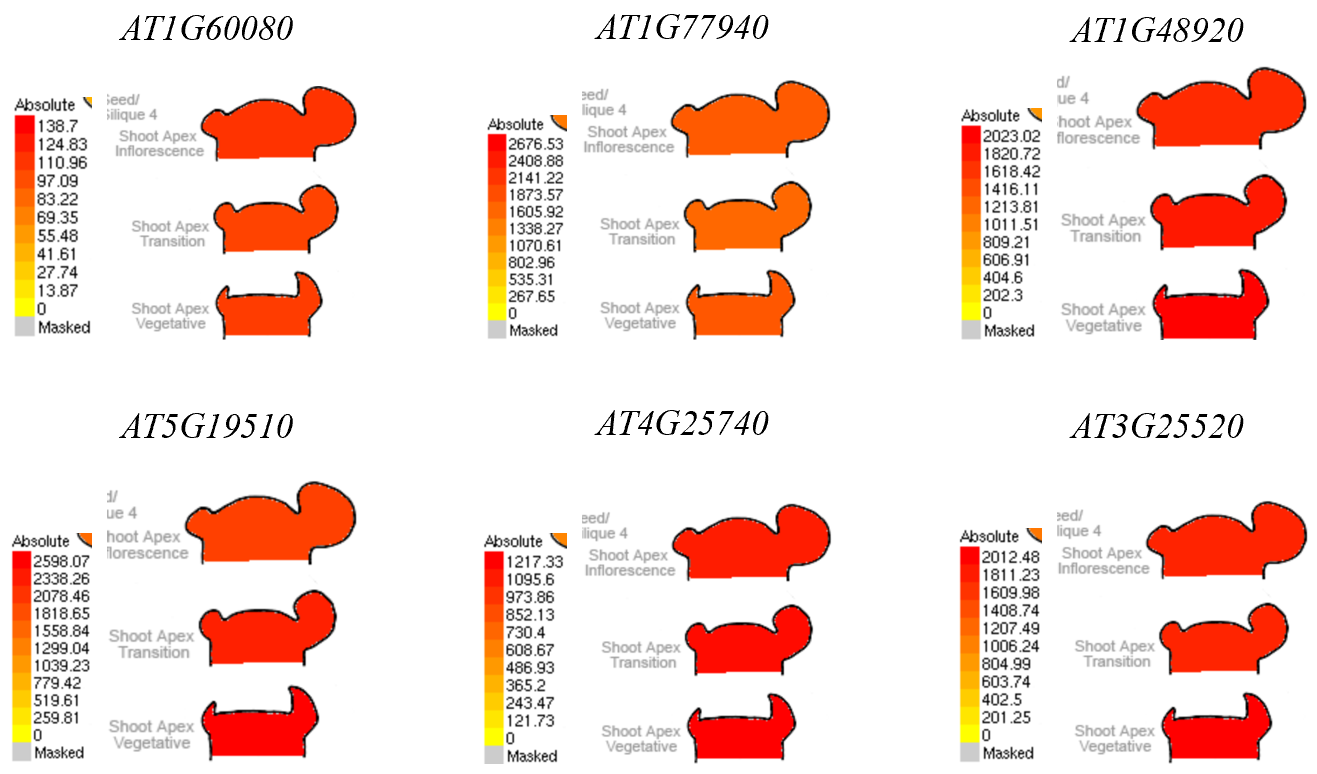
**

**
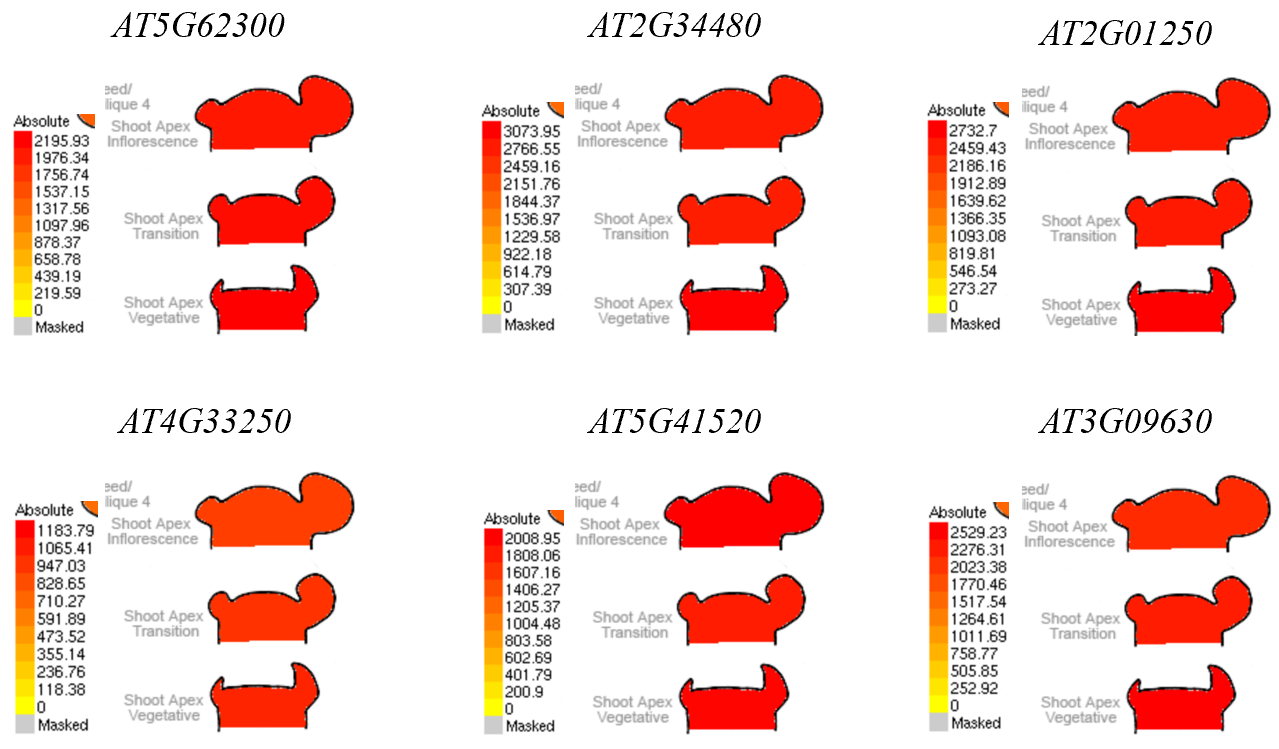
**

**
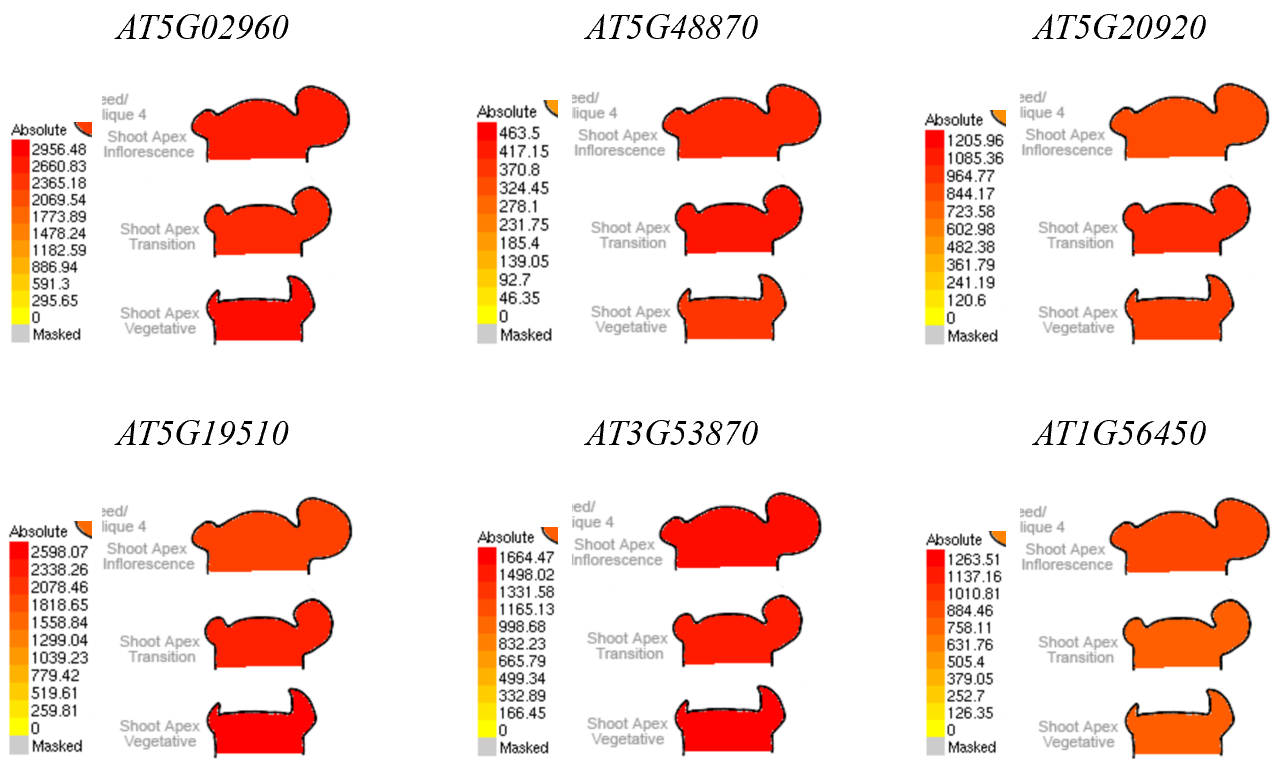
**

**Supplementary Figure S1.** The snapshot of eFP browser results showing some of methDEGs that are highly expressed in shoot apex in *Arabidopsis* (http://bar.utoronto.ca/efp/cgi-bin/efpWeb.cgi)


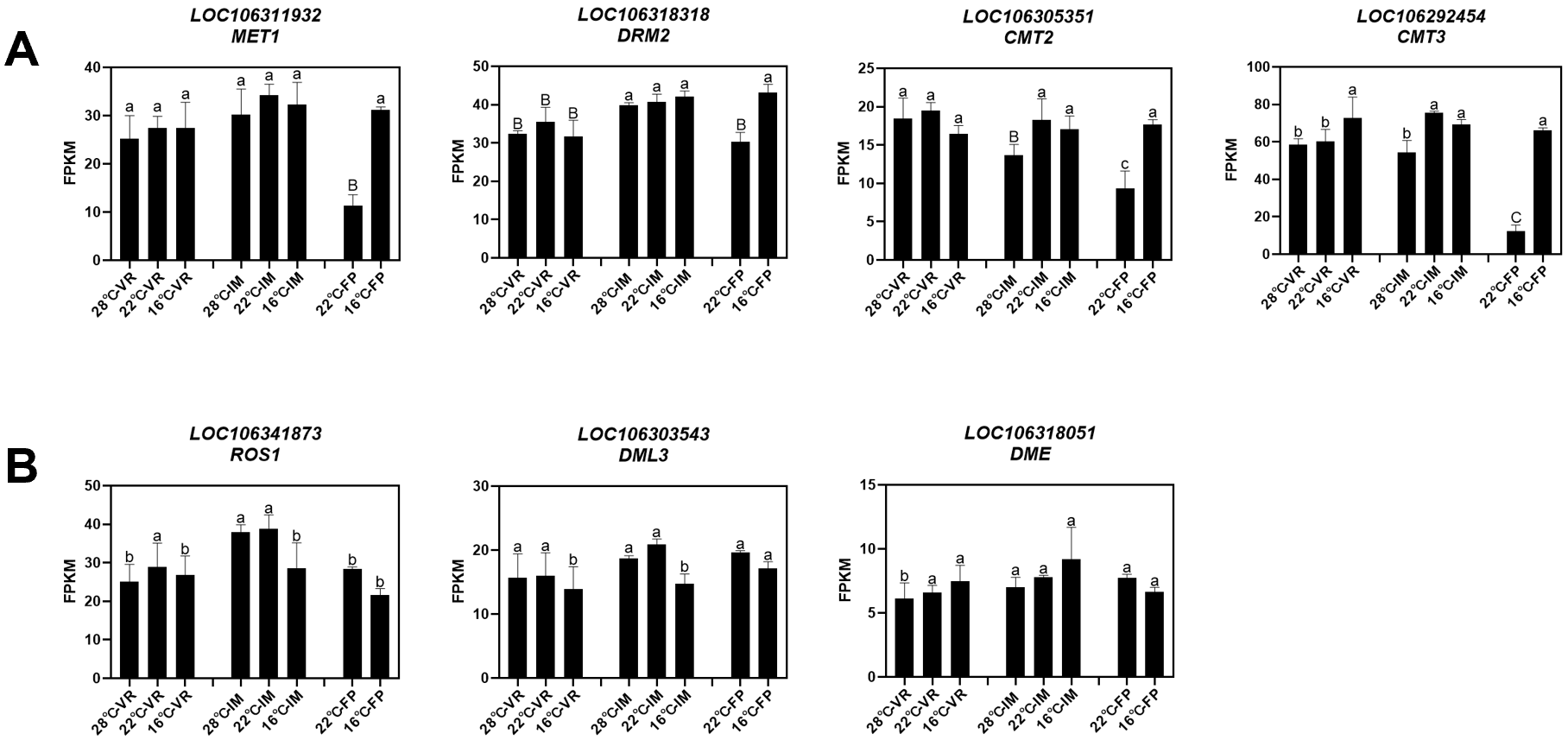


**Supplementary Figure S2.** Transcript levels of genes encoding DNA methyltransferases and demethylases in broccoli at three temperature regimes.

The expression of DNA methyltransferase-related genes (A) and demethylase-related genes (B) in broccoli apexes at VR, IM and FP stages under different temperature regimes as indicated. Data are mean values ± SD of transcript abundance from three related transcriptomes. Significant differences between means are indicated by different letters (lower letter indicates *P*-value < 0.05, capital letter indicates *P*-value < 0.01). ANOVA analysis with Dunnett’s test was used.

**Supplementary Table S1. Details of** **bisulfite sequencing libraries**

| Sample name | Clean Reads | Mapped reads | Unique Mapping Rate (%) | BS Conversion Rate (%) |
| --- | --- | --- | --- | --- |
| 28℃_VR_1 | 65219239 | 35394481 | 54.27 | 99.352 |
| 28℃_VR_2 | 63475785 | 34441960 | 54.26 | 99.399 |
| 28℃_IM_1 | 67713729 | 37019095 | 54.67 | 99.346 |
| 28℃_IM_2 | 62288896 | 33716979 | 54.13 | 99.367 |
| 22℃_VR_1 | 63446888 | 34153459 | 53.83 | 99.59 |
| 22℃_VR_2 | 62181736 | 33683846 | 54.17 | 99.615 |
| 22℃_IM_1 | 63784851 | 34660688 | 54.34 | 99.6 |
| 22℃_IM_2 | 63650781 | 34708770 | 54.53 | 99.599 |
| 22℃_FP_1 | 64444544 | 35496054 | 55.08 | 99.576 |
| 22℃_FP_2 | 62952548 | 34321729 | 54.52 | 99.588 |
| 16℃_VR_1 | 63580936 | 33844132 | 53.23 | 99.343 |
| 16℃_VR_2 | 62150427 | 33244263 | 53.49 | 99.419 |
| 16℃_IM_1 | 63580091 | 34352323 | 54.03 | 99.37 |
| 16℃_IM_2 | 67401030 | 36275234 | 53.82 | 99.257 |
| 16℃_FP_1 | 63394136 | 34188457 | 53.93 | 99.341 |
| 16℃_FP_2 | 67280387 | 36519794 | 54.28 | 99.381 |
| 16℃_FB_1 | 78446896 | 42847694 | 54.62 | 99.417 |
| 16℃_FB_2 | 63185118 | 34277926 | 54.25 | 99.402 |

**Supplementary Table S2. The methylation density of whole genome**

| Sample name | C | mC | mC  percent (%) | mCG percent (%) | mCHG percent (%) | mCHH  percent (%) |
| --- | --- | --- | --- | --- | --- | --- |
| 28℃_VR_1 | 160991676 | 17757800 | 11.03 | 30.63 | 22.61 | 4.56 |
| 28℃_VR_2 | 160991676 | 17614808 | 10.94 | 30.62 | 22.39 | 4.49 |
| 28℃_IM_1 | 160991676 | 18300402 | 11.36 | 32.19 | 24.99 | 4.23 |
| 28℃_IM_2 | 160991676 | 17406435 | 10.81 | 31.13 | 23.49 | 3.97 |
| 22℃_VR_1 | 160991676 | 16894108 | 10.49 | 29.67 | 21.44 | 4.25 |
| 22℃_VR_2 | 160991676 | 16684824 | 10.36 | 29.16 | 21 | 4.26 |
| 22℃_IM_1 | 160991676 | 16036460 | 9.96 | 28.96 | 20.77 | 3.78 |
| 22℃_IM_2 | 160991676 | 16635712 | 10.33 | 30.20 | 21.32 | 3.93 |
| 22℃_FP_1 | 160991676 | 16312583 | 10.13 | 28.82 | 20.52 | 4.10 |
| 22℃_FP_2 | 160991676 | 16821538 | 10.44 | 30.20 | 21.97 | 3.96 |
| 16℃_VR_1 | 160991676 | 16271085 | 10.10 | 30.18 | 21.18 | 3.65 |
| 16℃_VR_2 | 160991676 | 16165831 | 10.04 | 29.65 | 21.11 | 3.68 |
| 16℃_IM_1 | 160991676 | 16389925 | 9.61 | 30.57 | 21.22 | 2.85 |
| 16℃_IM_2 | 160991676 | 15878190 | 9.98 | 32.78 | 22.69 | 2.62 |
| 16℃_FP_1 | 160991676 | 15743674 | 9.77 | 31.06 | 21.28 | 2.98 |
| 16℃_FP_2 | 160991676 | 16417877 | 10.19 | 31.37 | 21.97 | 3.36 |
| 16℃_FB_1 | 160991676 | 18771426 | 11.65 | 35.06 | 23.86 | 4.28 |
| 16℃_FB_2 | 160991676 | 16155813 | 10.03 | 29.94 | 20.23 | 3.79 |

**Supplementary Table S3. Methylation levels of whole-genome**

| Sample name | | MeanC (%) | | MeanCG (%) | MeanCHG (%) | MeanCHH (%) |
| --- | --- | --- | --- | --- | --- | --- |
| 28℃_VR_1 | | 14.45 | 51.33 | 27.57 | 4.5 |  |
| 28℃_VR_2 | | 14.25 | 50.67 | 26.81 | 4.39 |  |
| 28℃_IM_1 | | 14.57 | 52.03 | 29.85 | 4.08 |  |
| 28℃_IM_2 | | 14.56 | 52.5 | 28.79 | 4.05 |  |
| 22℃_VR_1 | | 13.4 | 48.99 | 25.49 | 3.93 |  |
| 22℃_VR_2 | | 13.17 | 48.62 | 25.01 | 3.88 |  |
| 22℃_IM_1 | | 12.93 | 49.15 | 25.06 | 3.52 |  |
| 22℃_IM_2 | | 13.26 | 49.72 | 24.78 | 3.66 |  |
| 22℃_FP_1 | | 13.63 | 51.79 | 25.89 | 3.94 |  |
| 22℃_FP_2 | | 13.28 | 51.56 | 26.04 | 3.53 |  |
| 16℃_VR_1 | | 13.22 | 49.45 | 24.46 | 3.83 |  |
| 16℃_VR_2 | | 13.12 | 48.96 | 24.5 | 3.72 |  |
| 16℃_IM_1 | | 12.9 | 50.34 | 24.21 | 3.18 |  |
| 16℃_IM_2 | | 12.97 | 50.64 | 24.42 | 3.24 |  |
| 16℃_FP_1 | | 12.84 | 50.28 | 23.79 | 3.32 |  |
| 16℃_FP_2 | | 13.04 | 50.02 | 24.32 | 3.42 |  |
| 16℃_FB_1 | | 13 | 51.53 | 23.29 | 3.53 |  |
| 16℃_FB_2 | | 13.06 | 50.83 | 23.21 | 3.78 |  |

**Supplementary Table S4.** **Data from RNA-sequencing**

| Sample name | Raw Reads | Clean Reads | Clean Mapping rate (%) | Unique Reads | Unique Mapping rate(%) |
| --- | --- | --- | --- | --- | --- |
| 28℃_VR_1 | 41215222 | 35980497 | 87.30 | 35094775 | 85.15 |
| 28℃_VR_2 | 46399976 | 40908883 | 88.17 | 39881346 | 85.95 |
| 28℃_VR_3 | 41073514 | 36258834 | 88.28 | 35415444 | 86.22 |
| 28℃_IM_1 | 44489090 | 39578342 | 88.96 | 38594165 | 86.75 |
| 28℃_IM_2 | 42429132 | 37770904 | 89.02 | 36809632 | 86.76 |
| 28℃_IM_3 | 48466114 | 43186729 | 89.11 | 42119524 | 86.91 |
| 22℃_VR_1 | 43769272 | 38569590 | 88.12 | 37591063 | 85.88 |
| 22℃_VR_2 | 44622112 | 39637385 | 88.83 | 38634978 | 86.58 |
| 22℃_VR_3 | 47265686 | 42591014 | 90.11 | 41500498 | 87.80 |
| 22℃_IM_1 | 39586808 | 35431216 | 89.50 | 34533023 | 87.23 |
| 22℃_IM_2 | 41433476 | 37138725 | 89.63 | 36145696 | 87.24 |
| 22℃_IM_3 | 46076762 | 41413355 | 89.88 | 40332514 | 87.53 |
| 22℃_FP_1 | 46421040 | 41598639 | 89.61 | 40334568 | 86.89 |
| 22℃_FP_2 | 44423332 | 39776220 | 89.54 | 38566818 | 86.82 |
| 22℃_FP_3 | 42872026 | 38185440 | 89.07 | 37031362 | 86.38 |
| 16℃_VR_1 | 46511172 | 41812019 | 89.90 | 40634646 | 87.37 |
| 16℃_VR_2 | 40282582 | 36380077 | 90.31 | 35358124 | 87.78 |
| 16℃_VR_3 | 43443904 | 38693996 | 89.07 | 37642849 | 86.65 |
| 16℃_IM_1 | 47966892 | 43405585 | 90.49 | 40288385 | 83.99 |
| 16℃_IM_2 | 43932432 | 39245597 | 89.33 | 37716133 | 85.85 |
| 16℃_IM_3 | 46033156 | 41341207 | 89.81 | 39234201 | 85.23 |
| 16℃_FP_1 | 45393468 | 40466691 | 89.15 | 39351644 | 86.69 |
| 16℃_FP_2 | 46416132 | 41458397 | 89.32 | 40344951 | 86.92 |
| 16℃_FP_3 | 46120380 | 41213022 | 89.36 | 40080535 | 86.90 |
| 16℃_FB_1 | 46843092 | 41700422 | 89.02 | 40326278 | 86.09 |
| 16℃_FB_2 | 45119842 | 40335057 | 89.40 | 38994056 | 86.42 |
| 16℃_FB_3 | 47780180 | 42914643 | 89.82 | 41540809 | 86.94 |

**Supplementary Table S5. List of floral development cessation-associated genes (*FCG*s)**

| Floral Arrest Stage | | Gene Name | Gene Description |
| --- | --- | --- | --- |
| IM | *LOC106323250* | | P-loop containing nucleoside triphosphate hydrolases superfamily protein |
|  | *LOC106331660* | | RNA-binding (RRM/RBD/RNP motifs) family protein |
|  | *LOC106315696* | | exosome complex component RRP43%2C transcript variant X2 |
|  | *LOC106299001* | | Ribosomal protein L7Ae/L30e/S12e/Gadd45 family protein |
|  | *LOC106313474* | | Translation elongation factor EF1B/ribosomal protein S6 family protein |
|  | *LOC106301167* | | translation initiation factor 3 (IF-3) family protein |
|  | *LOC106315695* | | RNA binding Plectin/S10 domain-containing protein |
|  | *LOC106342956* | | exosome complex component RRP43%2C transcript variant X2 |
|  | *LOC106335414* | | 60S ribosomal protein L5-1 |
|  | *LOC106324940* | | 40S ribosomal protein S20-1 |
|  | *LOC106294921* | | 60S ribosomal protein L18a-2 |
|  | *LOC106319186* | | Cytosolic ribosomal 60S subunit protein |
|  | *LOC106331732* | | Encodes initiation factor 3k (EIF3k) |
|  | *LOC106300910* | | 60S ribosomal protein L30-2 |
|  | *LOC106298457* | | 40S ribosomal protein S10-2 |
|  | *LOC106336129* | | 60S ribosomal protein L4-1-like |
| FP | *LOC106300780* | | DNA-damage-repair/toleration protein DRT100-like |
|  | *LOC106336011* | | Ribosomal protein S12/S23 family protein |
|  | *LOC106331579* | | Ribosome associated membrane protein RAMP4 |
|  | *LOC106307354* | | 50S ribosomal protein L18%2C chloroplastic |
|  | *LOC106323568* | | polygalacturonase inhibitor 1-like |
|  | *LOC106316876* | | Sm-like snRNP proteins that are required for mRNA splicing, export, and degradation |
|  | *LOC106313474* | | Translation elongation factor EF1B/ribosomal protein S6 family protein |
|  | *LOC106337101* | | 40S ribosomal protein S3-2-like |
|  | *LOC106292555* | | RNA-binding KH domain-containing protein |
|  | *LOC106343390* | | probable small nuclear ribonucleoprotein |
|  | *LOC106336342* | | heterogeneous nuclear ribonucleoprotein 1-like |
|  | *LOC106300707* | | RNA recognition motif. (a.k.a. RRM, RBD, or RNP domain) |
|  | *LOC106315720* | | protein synthesis initiation factor eIF2 beta |

**Supplementary Table S6.** **Primer sequences for qRT-PCR**

| Gene ID | Forward Primer | Reverse Primer |
| --- | --- | --- |
| *LOC106300910* | AGAAGGCACAAAGAAGGCGT | TGCCACGACCACGGAAATTA |
| *LOC106336129* | TGAGAAGACTAACGCCGCAA | TTAGCTCCCTCGGTTCCGTA |
| *LOC106294921* | GGGCCACTAATGAAGTTCGC | ACGGAGCCAGATACCGTAGT |
| *LOC106301167* | GACCGAGGTCCAAATCAGCA | ACTCCAAACCGACCAGTAGC |
| *LOC106307354* | GCCAAGGCTGTGCGTATTTC | TCTTCGCCACCTCAATGGTT |
| *LOC106292555* | CCCTCAAGAAAGCGTGGCTA | CGGGTCTTGAGCTCGACTTT |
| *LOC106343390* | ACTTTGCACTATCGGTAACCATCT | AAGAGGAACTTGGTCGAGGT |
| *LOC106300707* | CGAGTCTTGGTCATGTCTTCAC | GCTTTTGACATTTCCGATGGCT |
| *Actin* | CCAGAGGTCTTGTTCCAGCCATC | GTTCCACCACTGAGCACAATGTTAC |
